# Current and New Approaches to Predict the Deflections of One-Way Flexural Members with a Focus on Composite Steel Deck Slabs Voided by Circular Tubes

**DOI:** 10.3390/ma14020421

**Published:** 2021-01-16

**Authors:** Chang-Hwan Lee, Iman Mansouri, Jaehoon Bae, Jaeho Ryu

**Affiliations:** 1 Division of Architectural and Fire Protection Engineering, Pukyong National University, 45, Yongso-ro, Nam-gu, Busan 48513, Korea; chlee@pknu.ac.kr; 2 Department of Civil Engineering, Birjand University of Technology, Ebne Hessam Square, Sanat & Madan Blvd., Birjand 97175-569, Iran; mansouri@birjandut.ac.ir; 3 School of Civil, Environmental, and Architectural Engineering, Korea University, 145, Anam-ro, Seongbuk-gu, Seoul 02841, Korea; skycitybjh80@gmail.com; 4 Technical Research Center, TechSquare Co., Ltd., 1, Gangnam-Daero 51-gil, Seocho-gu, Seoul 06628, Korea

**Keywords:** deflection, voided slab, composite slab, serviceability, effective moment of inertia, reinforcement ratio, load level, neutral axis

## Abstract

A new type of composite voided slab, the TUBEDECK (TD), which utilizes the structural function of profiled steel decks, has recently been proposed. Previous studies have confirmed that the flexural strength of TD slabs can be calculated based on the full composite contribution of the steel deck, but for long-span flexural members, the deflection serviceability requirement is often dominant. Herein, we derived a novel deflection prediction approach using the results of flexural tests on slab specimens, focusing on TD slabs. First, deflection prediction based on modifications of the current code was proposed. Results revealed that TD slabs exhibited smaller long-term deflections and at least 10% longer maximum span lengths than solid slabs, indicating their greater efficiency. Second, a novel rational method was derived for predicting deflections without computing the effective moment of inertia. The ultimate deflections predicted by the proposed method correlated closely with the deflection under maximum bending moments. To calculate immediate deflections, variation functions for the concrete strain at the extreme compression fiber and neutral axis depth were assumed with predictions in good agreement with experiments. The proposed procedure has important implications in highlighting a new perspective on the deflection prediction of reinforced concrete and composite flexural members.

## 1. Introduction

Compared with structural steel, reinforced concrete (RC) is traditionally considered to be unfavorable in long-span floor systems because of its small modulus of elasticity and large proportion of self-weight in the design load [1,2]. With this in mind, voided (V) slabs were designed to compensate for these shortcomings; in these slabs, the concrete volume of the section with a low contribution to flexural resistance is eliminated, thereby reducing weight [3,4]. To increase the structural and constructional merits, V slabs have been developed and applied in various forms over the past several decades [5,6,7,8].

**Figure 1:**
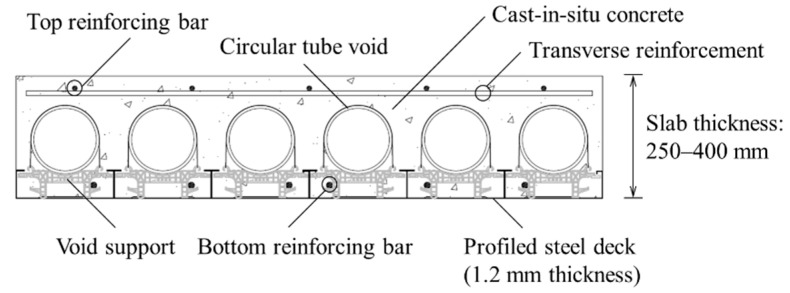
Typical cross-section configuration of a TUBEDECK (TD) slab.

A new type of V slab—called the TUBEDECK (TD)—was recently proposed (Figure 1) [9]. TD is a composite steel deck slab in which profiled steel decks, previously serving only as formwork, were utilized for structural purposes. Moreover, TD slabs are one-way flexural members that are voided by circular paper tubes. To evaluate the structural behavior of concrete and steel decks with T-shaped ribs and embossments on the bottom plate, a series of experimental studies were performed on TD slabs under various conditions [9,10]. From this, it was confirmed that the flexural strength based on full composite action could be reached without a mechanical device, and shear strength prediction equations and a moment–shear interaction design model were also proposed.

Sufficient strength for the ultimate state is an essential problem that must be overcome to prevent structural collapse, but serviceability performance is also of significance for the structure to function as intended. In particular, for RC or composite flexural members that cover long spans, such as TD slabs, satisfying the serviceability requirements related to deflections is often the most critical factor in design. Design codes for structural concrete [11,12] provide minimum thickness (or depth) that does not require deflection calculation, depending on boundary conditions and member type. For flexural members with a smaller thickness (or depth), the deflection needs to be calculated and checked to see if it complies with permissible deflections. However, if minimum thickness requirements for RC are applied to composite voided slabs such as TD slabs, excessive thickness may be required, which negate the advantage of flexural strength obtained by the composite action.

In particular, many concrete design codes provide a method for calculating deflection elastically using the effective moment of inertia (*I_e_*) [11,12,13]. Deflection prediction based on the semi-empirically derived *I_e_* (i.e., Branson equation) [14,15] has been found to work well for flexural members with a tension reinforcement ratio (*ρ*) between 1% and 2% [16,17]. Conversely, this method underestimates the deflection for members with *ρ* of less than 1%; hence, many studies have been conducted to derive *I_e_* in other forms [16,17,18,19,20,21]. Until now, such studies have mainly focused on fiber-reinforced polymer RC members. As the TD slabs considered in this study include structural steel decks as well as voids, it is unclear whether the existing deflection prediction methods for general RC flexural members are effective and economical.

With this background, this paper presents research performed on the derivation of a new deflection prediction approach, focusing on TD slabs—i.e., composite voided slabs. Details of previously performed experimental tests [9] used in this study are described, as are deflection prediction conditions for serviceability. As a first approach, deflection prediction methods based on the current code modification are introduced, and the deflection performance of TD slabs is evaluated through computational examples. As a second approach, a new method is derived that predicts deflection in a more rational way without the calculation of *I_e_*. Using this method, the ultimate deflections and immediate deflections are calculated, and the predicted values are compared with the experimental results. The proposed method is demonstrated to be effective in predicting the deflection of TD slabs. Finally, the significance and limitations of the proposed approach are presented.

## 2. Experimental Tests and Conditions for Deflection Prediction

### 2.1. Experimental Tests

**Figure 2:**
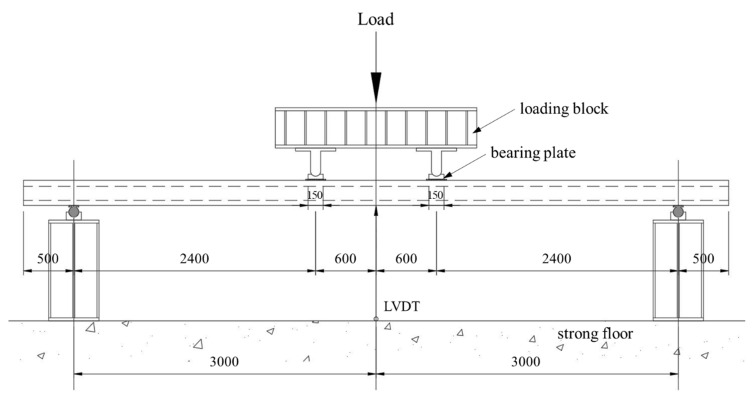
Experimental setup (all dimensions in mm).

**Figure 3:**
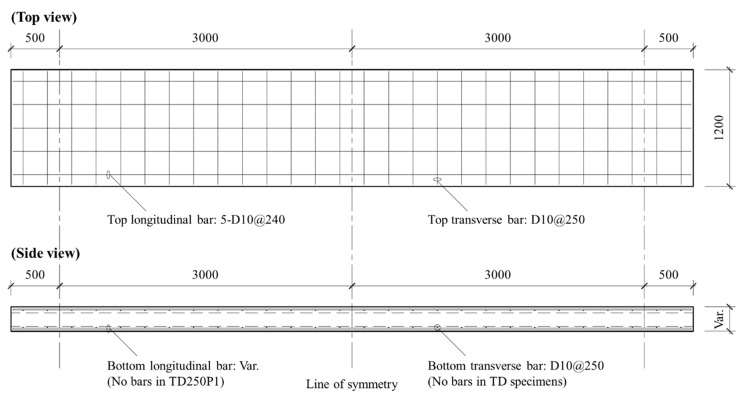
Reinforcement details of slab specimens (all dimensions in mm).

In this study, the deflection was evaluated on slab specimens tested by Lee et al. [9]. The experimental setup and general reinforcement details of the specimens are shown in Figure 2 and Figure 3, respectively. The slabs had a width of 1.2 m and a total length of 7.0 m. They were simply supported with a span length (*l*) of 6.0 m, and both sides had an overhang of 0.5 m. A two-point load was applied monotonically in the middle of the span so that the shear span length (*a_v_*) to each support point was 2.4 m. To prevent localized concrete damage, bearing plates were placed under both loading points. The experimental deflection was measured by a linear variable differential transducer (LVDT) installed under the specimen at the center of the span.

**Figure 4:**
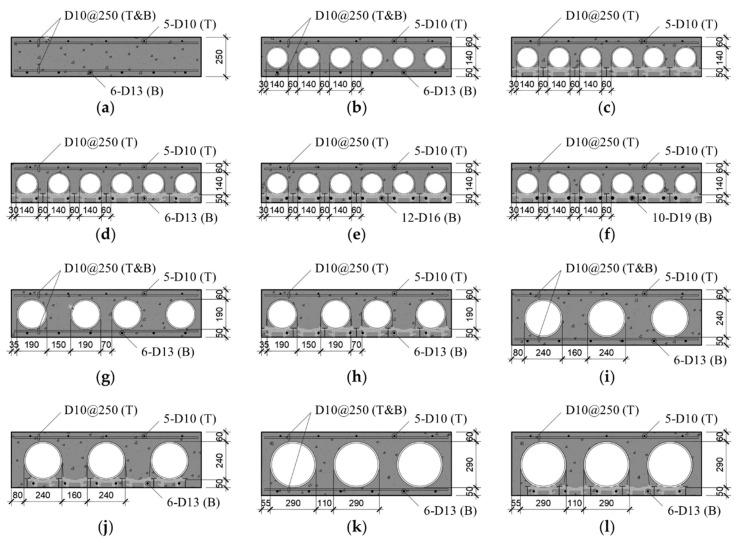
Cross-section details of specimens (all dimensions in mm): (**a**) S250P; (**b**) V250P; (**c**) TD250P1; (**d**) TD250P2; (**e**) TD250P3; (**f**) TD250P4; (**g**) V300P; (**h**) TD300P; (**i**) V350P; (**j**) TD350P; (**k**) V400P; (**l**) TD400P.

Table 1 presents a list of the tested slab specimens under consideration, and the cross-section details are shown in Figure 4. A total of 12 specimens were analyzed, consisting of one RC solid slab (S), four RC voided slabs (V), and TD slabs. The slab thickness (*t_s_*) ranged from 250 to 400 mm. The external diameter of the circular paper tube (*D_e_*) was designed to be 110 mm smaller than *t_s_*, and the number of voids (*n_v_*) was planned by considering *t_s_* and *D_e_*. The top longitudinal reinforcement for all slabs was the same: 5-D10@240 (i.e., 5 10 mm diameter bars spaced at 240 mm). The transverse reinforcement was constructed as D10@250 for both top and bottom but not at the bottom in TD slabs owing to the presence of the steel decks. V slabs and four TD slabs had *t_s_* as a variable (i.e., TD250P2, TD300P, TD350P, and TD400P) and had the same steel reinforcement, whereas their void ratios (*R_v_*) were 30.79%, 31.50%, 32.31%, and 41.28%, respectively. TD250P1 only had a steel deck with no reinforcing bar at the bottom. TD250P3 and TD250P4 had very heavy steel reinforcements that were intentionally designed to make the depth of the equivalent rectangular compressive stress block (*a*) larger than the thickness of the topping concrete (*t_ft_*).

**Figure 5:**
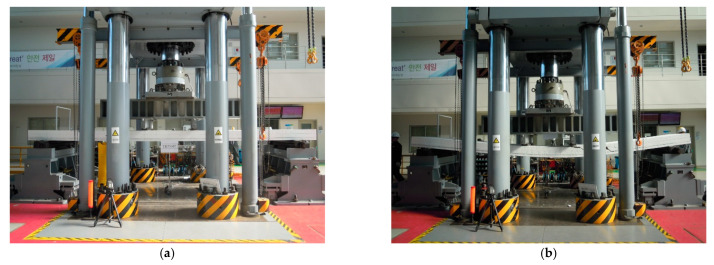
Photos of experimental tests: (**a**) before the loading test; (**b**) after failure.

The compressive strength of concrete ($f_{c}^{\prime }$) is presented in Table 1. The yield stresses of the reinforcing bars (*f_y_*) were 459.9, 440.4, 441.5, and 515.5 MPa for D10, D13, D16, and D19, respectively. Moreover, the yield stress of the steel deck (*F_y_*) with a thickness of 1.2 mm was 292.0 MPa. Figure 5 presents photographs of the slab specimen flexural test. The deflection (*δ*) was measured at midspan. The shear span-to-depth ratio was at least six or more for all specimens, indicating that the shear deformation component was negligible.

Before the loading test, the self-weight and weight of the bearing plates under the loading points (0.5 kN for each) were applied to the specimens. The experimental design was created with TD slabs as the main consideration. S and V slabs were reinforced with the same reinforcing bars as the comparative TD slabs mentioned earlier, and thus, they corresponded to very under-reinforced slabs. For this reason, their *M_pre_*/*M_n_* ratios were quite high, with a value of 0.450 for the S slab and a range of 0.252–0.318 for the V slabs (Table 1). Their *M_pre_* values were calculated to be smaller than their cracking moments, but several initial cracks were observed, which were more severe with a smaller *t_s_*. Conversely, the *M_pre_*/*M_n_* values were less than 0.1 for TD slabs except for TD250P1, and no visible cracks were observed for TD slabs before the loading test. As initial deflection caused by preloading (i.e., the self-weight and weight of the bearing plates) could not be measured, the results for deflection are compared after the *M_pre_*/*M_n_* values of the specimens in Section 3 and Section 4. In addition, the calculated values were used for the initial experimental deflections at the beginning of loading.

### 2.2. Conditions for Deflection Prediction

In concrete, in addition to the immediate deflection (*δ_i_*) owing to the direct action of the load, additional long-term deflection (*δ_cp_*_+*sh*_) occurs owing to creep and shrinkage. The deflection *δ_cp_*_+*sh*_ is obtained by multiplying the immediate deflection caused by the sustained load considered (*δ_sus_*) by a factor (*λ_δ_*) (Equation (1)), where *λ_δ_* is affected by the compression reinforcement ratio ($\rho^{\prime }$) and the time-dependent factor (*ξ*), as in Equation (2) [11,14].

(1) $$\delta_{cp+sh}=\lambda_{\delta }\delta_{sus}$$

(2) $$\lambda_{\delta }=\frac{\xi }{1+50\rho^{\prime }}\;$$

The sustained load is considered by adding some portion of live load (*L*) to the dead load (*D*). The ratio of *L* added to *D* varies depending on usage, but usually less than 50% of *L* is considered for this purpose [23]. However, because the stiffness of the section may change owing to cracking, the immediate deflection because of *L* (*δ_L_*) must be calculated by subtracting the immediate deflection owing to *D* (*δ_D_*) from the deflection caused by a combination of *D* and *L* (*δ_D_*_+*L*_), as in Equation (3). Therefore, a deflection prediction equation should theoretically be able to predict the *δ_i_* effectively at a service load level between 1.0*D* and 1.0*D* + 1.0*L*.

(3) $$\delta_{L}=\delta_{D+L}-\delta_{D}$$

The primary use of TD slabs is in parking garages. In this case, *L* is typically 6 kPa [12], and the self-weight (SW) can be calculated using a density of 24 kN/m^3^ for all specimens. The superimposed dead load (SDL), excluding the SW, was considered to be 2.5 kPa, including the 100 mm thick plain concrete and finishes such as the ceiling. Using these conditions, serviceability limit states (SLS) for deflection checks by each *t_s_* were classified into SLS 1 (1.0*D*), SLS 2 (1.0*D* + 0.5*L*), and SLS 3 (1.0*D* + 1.0*L*), as shown in Table 2. In the table, ULS indicates the load level of 1.2*D* + 1.6*L* for the strength limit state used in the design. As this study is based on an experiment, *M_n_* was used as a reference value in the analysis. However, when calculating the design strength of members, a strength reduction factor (*φ*) is applied, as in Equation (4):

(4) $$M_{u}\leq \varphi M_{n}$$

where *M_u_* denotes the factored bending moment. In ACI 318, the *φ* for tension-controlled sections is given as 0.9 [24], and this value was used in Table 2. For a parking garage, it is common to consider a sustained load at the level of SLS 2, but as mentioned above, the effective prediction range (i.e., *M*/*M_n_*) of the prediction equation for the *δ_i_* should be at least the entire range from SLS 1 to SLS 3. When designing a section, it is rare that rebars are placed to fit perfectly to the required moment capacity, and rebars may also be added for deflection control. Therefore, in practice, a certain range below SLS 1 should also be included in the effective prediction range. Based on this consideration, the effective *M*/*M_n_* range of the prediction equation for the slabs under consideration may be set between approximately 0.30–0.65.

## 3. Deflection Prediction Based on Modifications of the Current Code

### 3.1. State-of-the-Art Prediction Method

ACI 318 permits the calculation of the *δ_i_* elastically, using the modulus of elasticity of concrete (*E_c_*) and the effective moment of inertia (*I_e_*) for RC members. In the ACI 318-14 code, the *I_e_* is given by Equation (5) [11]:

(5) $$I_{e}={\left(\frac{M_{cr}}{M}\right)}^{3}I_{g}+\left[1-{\left(\frac{M_{cr}}{M}\right)}^{3}\right]I_{cr}$$

where *M* is the maximum bending moment in the member due to the service loads, and *M_cr_* is the cracking moment, which can be calculated using Equation (6). Moreover, *I_cr_* is the moment of inertia of cracked section transformed to concrete, and *I_e_* according to Equation (5) cannot be greater than *I_g_* (i.e., the moment of inertia of the gross concrete cross-section about the centroidal axis, neglecting steel reinforcement).

(6) $$M_{cr}=f_{r}I_{g}/y_{t}$$

In the above equation, *f_r_* denotes the modulus of rupture of the concrete, which can be calculated as $0.62\sqrt{f_{c}^{\prime }}$ ($0.6\sqrt{f_{c}^{\prime }}$ in the CSA A23.3 standard [13,25]) for normal-weight concrete, and *y_t_* is the distance from the centroidal axis of gross section, neglecting reinforcement, to the tension face.

The value of *I_e_* given by Equation (5) is based on the Branson equation derived using a semi-empirical approach [15]. Although this equation predicts the deflection of RC beams with a *ρ* between 1% and 2% well, it has been reported that the predicted deflections were smaller than the experimental results for beams and slabs with a *ρ* less than 1%. The problem of underestimation has been continuously raised [17,18], and as a result, the equation for calculating *I_e_* has recently been revised in the current ACI 318 [19,24]. According to the ACI 318-19 building code, when *M* is greater than (2/3) *M_cr_*, the *I_e_* is given by the following equation:

(7) $$I_{e}=\frac{I_{cr}}{1-{\left(\frac{\left(2/3\right)M_{cr}}{M}\right)}^{2}\left(1-\frac{I_{cr}}{I_{g}}\right)}$$

where *M_cr_* is computed by Equation (6), and when *M* is equal to or less than (2/3) *M_cr_*, *I_g_* is used as the value of *I_e_*.

The CSA A23.3 standard made modifications to reduce underestimation at a low *ρ* prior to the ACI 318 code. The CSA A23.3-14 standard calculates deflections using Equations (5) and (6) in the same way as the ACI 318-14 code but must compute *M_cr_* using 0.5*f_r_* instead of *f_r_* [13]. Through this, it compensates for the unconservativism owing to cracks caused by shrinkage or temperature effects, and this approach is also adopted in the current CSA A23.3-19 standard [25].

### 3.2. S Slab

**Figure 6:**
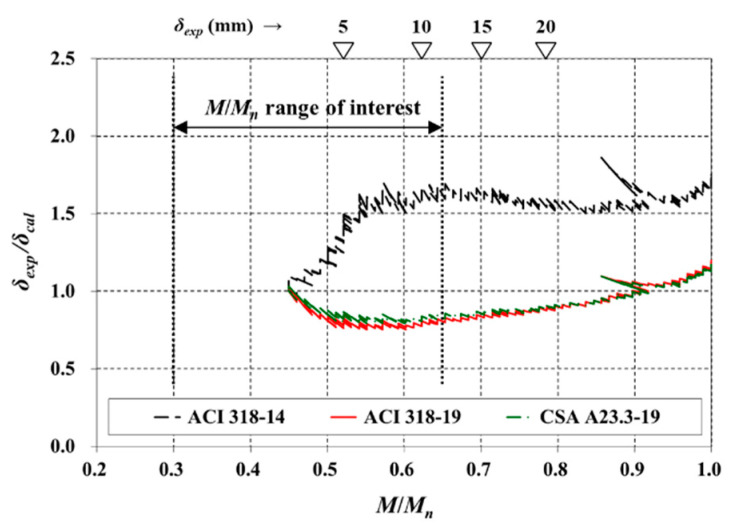
Comparison of immediate deflection (S slab).

Figure 6 compares the calculated deflection (*δ_cal_*) and experimental deflection at midspan (*δ_exp_*) for S250P, a solid slab with no voids. The *M_pre_*/*M_n_* for this specimen was 0.450 (Table 1). Accordingly, *δ_exp_*/*δ_cal_* could be calculated only in the range where *M*/*M_n_* was greater than 0.450, and the initial value of *δ_exp_*/*δ_cal_* was normalized to 1.0 by the deflection owing to preloading (*δ_pre_*) (see Section 2.1).

The bottom reinforcement of S250P was 6-D13, and the *ρ* was relatively small with a value of 0.283%. The ACI 318-14 code, which uses Equations (5) and (6) in predicting the *δ_cal_*, significantly underestimates deflection, similarly to previous studies [16,17,18,19,20]. Conversely, the deflection calculated using the ACI 318-19 code yielded improved results. The CSA A23.3-19 standard, which is based on 0.5*f_r_* (i.e., 0.5*M_cr_*) and Equation (5), also predicted the deflection of S250P relatively well in the range of interest.

### 3.3. V Slabs

**Figure 7:**
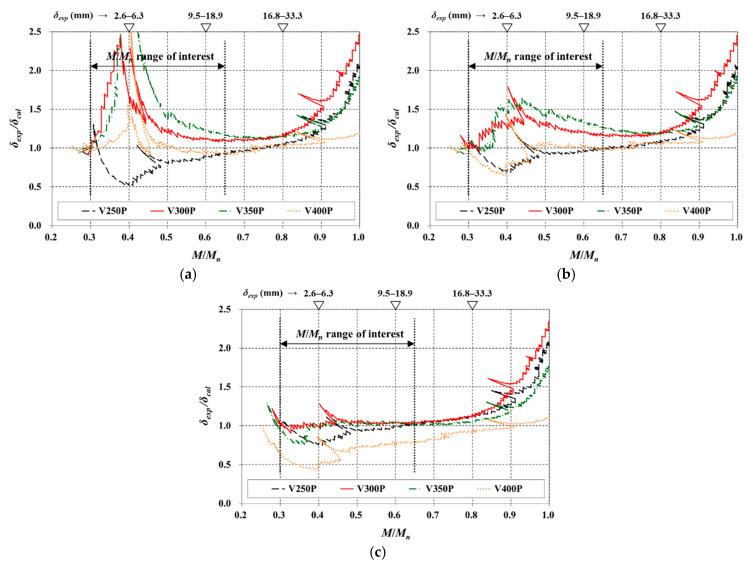
Comparison of immediate deflection (V slabs) based on (**a**) ACI 318-19; (**b**) CSA A23.3-19 (0.5*f_r_*); (**c**) 0.35*f_r_*.

The comparisons for the four V slabs are shown in Figure 7. The slabs were very lightly reinforced where the *ρ* values ranged 0.170–0.283%. For this reason, the deflection according to the previous ACI 318-14 code was predicted to be very small compared to the *δ_exp_* over the entire range of *M*/*M_n_* (results are not shown in Figure 7). In the case of the ACI 318-19 code and the CSA A23.3-19 standard, the *δ_cal_* was at an acceptable level in the *M*/*M_n_* range of interest for V250P and V400P, whereas the *δ_exp_*/*δ_cal_* was significantly greater than 1 for V300P and V350P (Figure 7a,b). In other words, the prediction equations significantly underestimated the deflection of V300P and V350P, which is primarily because the stiffnesses of the members were largely overestimated [17,18].

When computing *I_g_* of the V slabs, a stiffness reduction owing to voids had already been considered. However, it should be understood that it alone was insufficient to reflect the actual stiffness of the V slabs. In addition, it is presumed that the presence of voids itself increased the stiffness reduction. As V slabs with a low *ρ* are more prone to cracking owing to shrinkage and temperature effects, additional considerations are required to avoid unconservative deflection predictions.

Comparing Figure 7a,b, the CSA A23.3-19 standard yielded more stable results than the ACI 318-19 code, especially for the low *M*/*M_n_* region. Thus, deflection calculations were made for V slabs with additional modifications to the CSA A23.3-19 standard (or ACI 318-14 code). Specifically, the *M_cr_* was calculated using 0.35*f_r_*, and the result is shown in Figure 7c. In addition, the values for the various parameters used in the immediate deflection calculation are summarized in Table 3, where it can be observed that the *I_g_*/*I_cr_* is considerably larger than the range in which the Branson equation works well (i.e., *I_g_*/*I_cr_* < 3) [16].

With the use of 0.35*f_r_*, the *δ_cal_* for V300P and V350P got very close to the *δ_exp_* in the range of interest. For V250P, there was some unconservativism when the *M*/*M_n_* ratio was less than 0.45 (the difference between the *δ_cal_* and *δ_exp_* was less than 4 mm), but overall, acceptable results were obtained. For V400P, the *δ_exp_*/*δ_cal_* was between 0.44–0.73 in the *M*/*M_n_* range of 0.3–0.5, and the value (*δ_exp_*/*δ_cal_*) was 0.80 at *M*/*M_n_* = 0.65—the upper limit of the range of interest. Although the *δ_exp_*/*δ_cal_* ratio tended to be significantly less than 1 over the range of interest, the maximum value of the difference (*δ_cal_*–*δ_exp_*) in that range was only 3.5 mm as it was a nonslender member with an *l*/*t_s_* ratio of 15. The modified equation yielded a different result depending on the *t_s_*, but this approach, which used a reduced factor for *f_r_*, could effectively reflect the stiffness reduction owing to the voids. Until further detailed experimental verification, based on *M_cr_* calculated using 0.35*f_r_*, the immediate deflection for V slabs can be predicted from a conservative perspective.

### 3.4. TD Slabs

**Figure 8:**
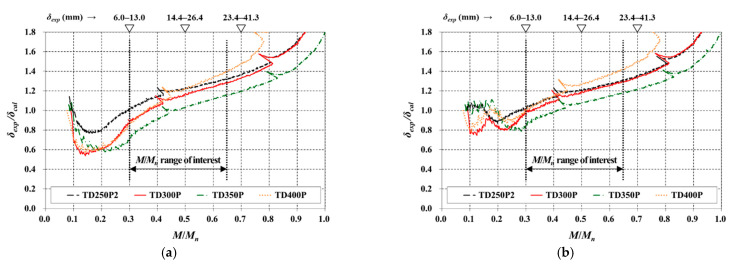

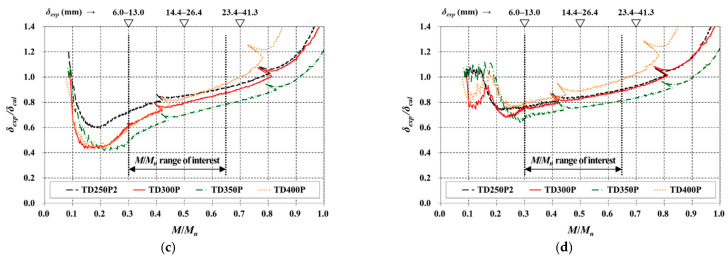
Comparison of immediate deflection (TD slabs with a variable of slab thickness): (**a**) 0.5*f_r_* and full contribution conditions; (**b**) *f_r_* and full contribution conditions; (**c**) 0.5*f_r_* and half contribution conditions; (**d**) *f_r_* and half contribution conditions.

Figure 8 compares the deflections for TD slabs with the same reinforcement and different *t_s_* (TD250P2, TD300P, TD350P, and TD400P). Because they had a high *ρ* compared with V slabs, the evaluations were made for cases using 0.5*f_r_* (CSA A23.3-19) and *f_r_* (ACI 318-14), without using 0.35*f_r_*. In Figure 8a,b, the entire area of the steel deck (*A_sd_*) was considered to contribute to the stiffness in the same way as the reinforcing bar. In these cases, it can be seen that the factor of the applied *f_r_* had a great influence in a low *M*/*M_n_* range, and that *δ_cal_* did not change significantly based on a factor of *f_r_* when the *M*/*M_n_* was greater than approximately 0.5. However, both methods resulted in a large underestimation of the deflection in the range of interest.

Lee et al. [9] reported that the contribution of the steel deck could be considered to be a full composite when evaluating the flexural strength of the TD slabs with an appropriate amount of tension reinforcement. However, the bottom plate of the steel deck inevitably has a lower contribution to the stiffness compared with a T-shaped rib or rebar completely embedded in the concrete (see Figure 1). As the effect of such a partial contribution of the steel deck to stiffness was not reflected, the deflection was predicted to be small in Figure 8a,b. As a practical method to compensate for this problem, half of the contribution of *A_sd_* was considered when calculating *I_cr_*; the results are shown in Figure 8c,d.

Under the half contribution condition, the *δ_exp_*/*δ_cal_* was less than 1 within the range of interest for both cases where *M_cr_* was computed using 0.5*f_r_* and *f_r_*; hence, both methods could be used to predict the immediate deflection from a conservative perspective. When 0.5*f_r_* was used, the predicted deflection exhibited a relatively large error in the range of *M*/*M_n_* of less than 0.4 (Figure 8c). Even when half of the contribution of *A_sd_* was considered, the maximum *I_g_*/*I_cr_* ratio was 2.892 (Table 4), which corresponded to a *ρ* of more than 1% [16]. Because of this, predictions based on Equations (5) and (6)—as in the ACI 318-14 code—would have yielded more reasonable results, as shown in Figure 8d. The value of *δ_exp_*/*δ_cal_* using this approach was the smallest at the lower limit (*M*/*M_n_* = 0.30) within the range of interest, with a range of 0.71–0.80. Excluding TD400P, where early failure occurred owing to inadequate void arrangement [9], the range of *δ_exp_*/*δ_cal_* at the upper limit (*M*/*M_n_* = 0.65) was 0.82–0.90, and, accordingly, it can be observed that the immediate deflection was calculated conservatively by at least 10%. Although the ratio could be further reduced by increasing the effective contributing area of the steel deck, a slightly conservative prediction would be desirable because the number and conditions of the specimens evaluated in this study were limited. Moreover, if the bending moment was the same, the uniform load condition caused approximately 5% larger elastic deflection than the loading situation considered in this study. Therefore, considering various uncertainties, the conservatism depicted in Figure 8d can be considered to be sufficiently reasonable.

As analyzed above, the *I_e_* equation (Equation (5)) given in recent concrete standards, i.e., CSA A23.3-19 and ACI 318-14, was also valid in calculating the immediate deflection of TD slabs. However, to not overestimate the flexural stiffness of the member, it was recommended that *I_cr_* be computed by considering only the half area of the steel deck as the effective contributing area. The suggested prediction method based on code modification can be practically used to calculate the immediate deflection of the TD slabs.

**Figure 9:**
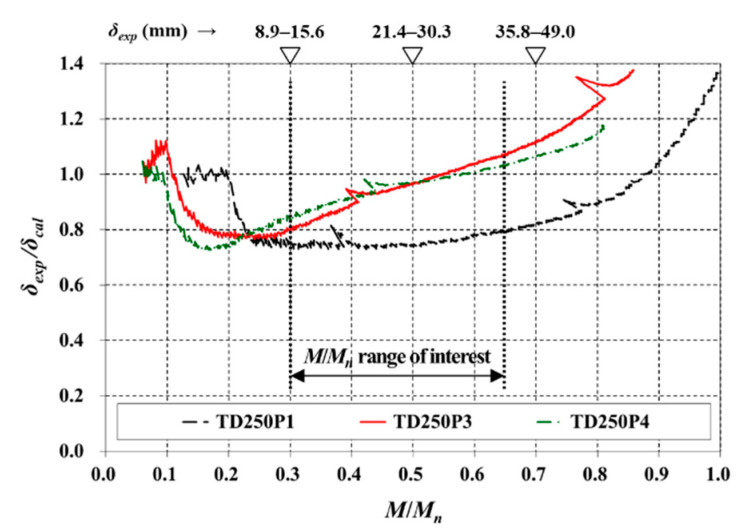
Comparison of immediate deflection (of other TD slabs with a variable of *ρ*).

The deflections of other TD slabs (TD250P1, TD250P3, and TD250P4) using the suggested method are compared in Figure 9. In the effective *M*/*M_n_* range, the *δ_exp_*/*δ_cal_* ratio of TD250P1 with no bottom reinforcing bar was less than 0.8, and the predicted deflection was conservative overall. TD250P3 and TD250P4 were designed with *a* > *t_ft_*, and their *δ_exp_*/*δ_cal_* ratios tended to increase steadily as *M*/*M_n_* increased. For *M*/*M_n_* greater than 0.55, the deflection was slightly underestimated. However, for ductile behavior and the safety against combined flexure and shear, designing *a* to be smaller than *t_ft_* has been recommended [9,10]. Therefore, the results of TD250P3 and TD250P4, which were very heavily reinforced intentionally, do not limit the applicability of the suggested equation.

Table 5 presents the *δ_exp_* value and deflection ratio (*l*/*δ_exp_*) of the TD slabs for each SLS, and an extremely unfavorable situation (*M_u_* = *φM_n_*) with no excess flexural strength assumed. The difference between SLS 3 and SLS 1 is the deflection corresponding to *δ_L_* given by Equation (3), and this value ranged from *l*/502–*l*/233. The sustained load of a parking garage is close to SLS 2 (1.0*D* + 0.5*L*); hence, it is possible to exceed the maximum permissible deflection in many slabs, considering the long-term effect. Therefore, when *l* is relatively large compared to *t_s_*, it should be designed with special attention to satisfying the deflection serviceability requirements.

### 3.5. Computation Examples

This section evaluates the comparative performance of S and TD slabs for immediate and long-term deflections using the prediction methods discussed above. Comparative computations are made for cases where the two types of slabs have the same flexural strength level and the same tension reinforcing bars. An SDL of 2.5 kPa and a live load of 6.0 kPa were applied as in Section 2.2, and the limitations for the *δ_L_* and *δ_cp_*_+*sh*_+*δ_L_* were set to *l*/360 and *l*/240, respectively.

#### 3.5.1. Condition 1: Slabs with the Same Strength Level

The simply supported one-way slabs were considered, and in a given condition, the maximum *l* was 20*t_s_*, unless deflections were computed [12,24]. In this example, starting with *l* = 20*t_s_*, the computations were repeated by increasing *l* in steps equal to *t_s_* until both slab systems did not satisfy permissible deflections. The sections were designed for the strength limit state prior to the deflection check, where the difference in *φM_n_*/*M_u_* was limited to less than 10%, such that the two slabs had similar flexural strength. The assumed values for *f′_c_*, *f_y_*, and *F_y_* were 24, 400, and 245 MPa, respectively, and the calculated deflections are summarized in Table 6.

Although the *ρ* values for all S slabs were less than 1.0%, 0.5*f_r_* introduced in Section 3.2 was not used to compute the *M_cr_*. In other words, the deflection was calculated based on Equations (5) and (6) to avoid providing relatively favorable results for TD slabs. A *ξ* value of 2.0 (5 years or more) was used, and the sustained load was considered to be SLS 2 (1.0*D*+0.5*L*). In the TD slabs with a *t_s_* of 250, 300, and 350 mm, only the steel deck exhibited sufficient flexural strength, and the tension reinforcement was the same even with increasing *l*. As the partial contribution to stiffness was considered for the steel deck (refer to Section 3.4), *I_cr_* of the TD slabs was less than 20% compared with that of the S slabs designed to the same strength level.

In all cases, *δ_L_* was significantly lower than the permissible value, and it tended to occur largely in TD slabs. By contrast, it is noteworthy that *δ_cp_*_+*sh*_ + *δ_L_* was predicted to be smaller in TD slabs for all cases. This suggests that the effect of the self-weight reduction in the TD slabs owing to voids was large enough to offset the increase in *δ_L_* (because of a decrease in stiffness related to *I_g_* and *I_cr_*). From this, it can also be explained that the TD slab system is more efficient in deflection control than S slabs.

#### 3.5.2. Condition 2: Slabs with the Same Tension Reinforcing Bars

To evaluate the contribution to deflection control owing to the use of steel decks, computations were performed for the TD slabs and S slabs with the same tension reinforcing bars. The minimum reinforcement and maximum *l* satisfying both the strength limit state and permissible deflections for the S slabs were first determined, followed by computations for the TD slabs with the same reinforcing bars. Then, within a range where *φM_n_*/*M_u_* of the S slab was less than 2, the maximum *l* was repeatedly computed by increasing reinforcing bars. Undescribed conditions were the same as in the previous example, and the results are shown in Table 7.

Under the given conditions, the maximum *ρ* of S slabs for all *t_s_* values was between 0.874% (*t_s_* = 400 mm) and 0.895% (*t_s_* = 250 mm). Owing to the contribution of the steel deck, *φM_n_*/*M_u_* exceeded 2 for all TD slabs, and their *I_cr_* also increased by 1.25–2.05 times compared with S slabs. The deflection *δ_L_* was significantly smaller than the permissible value, and the maximum *l* was determined by *δ_cp_*_+*sh*_ + *δ_L_*. The maximum *l* for S and TD slabs was calculated to be 20.0–22.2*t_s_* and 22.3–24.8*t_s_*, respectively. It tended to decrease gradually as *t_s_* increased, but in all cases, the maximum *l* of the TD slabs was at least 10% larger than that of S slabs.

## 4. Deflection Prediction Based on the New Rational Method

### 4.1. Concept of the Proposed Prediction Method

This section proposes a prediction method that combines structural mechanics and flexure theory for RC members to calculate deflection without computing the empirically derived *I_e_*. Based on a linear strain distribution and also under small deflection conditions, the concrete strain at the extreme compression fiber (*ε_c_*) and curvature (*κ*) have the relationship shown in Equation (8) [26,27]:

(8) $$\kappa =1/R=\epsilon_{c}/c$$

where *R* is the radius of curvature, and *c* is the distance from the extreme compression fiber to the neutral axis. According to classical elastic theory, the moment–curvature relationship is expressed by Equation (9) [28]:

(9) 1/R=M/EI

where the product, *EI*, is referred to as the flexural rigidity of the section. Combining Equations (8) and (9), *EI* is formulated as follows:

(10) $$EI=Mc/\epsilon_{c}$$

**Figure 10:**
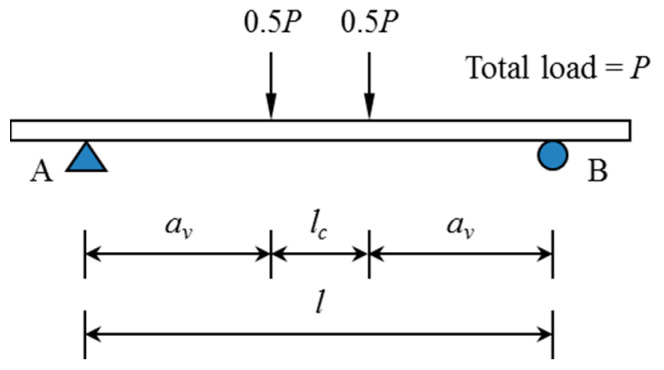
Imposed point loading condition in the experiment.

The imposed point loading condition of the specimens in this study can be expressed simply as shown in Figure 10. Assuming a linear elastic beam, the deflection *δ* at midspan is given by Equation (11). Then, when the flexural rigidity obtained using Equation (10) is substituted into Equation (11), a deflection prediction equation expressed by Equation (12) can be obtained:

(11) $$\delta =\frac{Pa_{v}}{48EI}\left(3l^{2}-4a_{v}{}^{2}\right)$$

(12) $$\delta =\frac{Pa_{v}}{48}\frac{\epsilon_{c}}{Mc}\left(3l^{2}-4a_{v}{}^{2}\right)$$

In Equation (12), *l* and *a_v_* are constant values given by loading conditions, whereas *ε_c_* and *c* are variables that change according to the load level. *P* and *M* are also values determined according to the load level. For tested specimens, *P* is given by the machine load, but a preloading effect must be added when calculating *M* (i.e., *M* = *M_pre_* + 0.5*Pa_v_*).

### 4.2. Prediction of Ultimate Deflections

Based on Equation (12), the ultimate deflections calculated using the *c* value (i.e., *c_u_*) corresponding to *M_n_* and the maximum usable concrete strain at an extreme compression fiber (*ε_cu_*) of 0.003 are summarized in Table 8. The *δ_cal_* predicted by the proposed method had a considerably larger error with the *δ_exp_* when *M_n_* was reached, but it was rather closely correlated with the displacement at the maximum bending moment (*M_max_*). During the experiment, early failure occurred without reaching *M_n_* in TD250P3 and TD250P4, which were intentionally very heavily reinforced and in TD400P, which was evaluated to have an inadequate void arrangement [9]. Excluding them, the *δ_exp_*/*δ_cal_* of the TD slabs at the *M_max_* ranged 1.098–1.142, and the proposed equation tended to predict the ultimate deflections very closely.

In the case of S250P and V250P, the experiment was terminated with a load level slightly below the peak strength owing to equipment problems. Nevertheless, their *δ_exp_* values were greater than the *δ_cal_*, and the ultimate deflection prediction was valid. The experimental ultimate deflection of V300P was less than the *δ_cal_*, though the *δ_exp_*/*δ_cal_* was 0.965, which was very close to 1. Conversely, the *δ_exp_*/*δ_cal_* values for V350P and V400P, which were thick V slabs, were quite small compared to 1. They failed by fracture of tension reinforcing bars with an increase in strain demand, which corresponded to cases where the values of *c* at the *M_max_* based on the measured strains differed significantly from the predicted values (calculated according to the code).

As such, the proposed approach was able to predict the ultimate deflections of the TD slabs with normal flexural failure fairly accurately. Although *δ_cal_* tended to be evaluated largely for thick slabs designed with an extremely low *ρ*, the results were well matched for slender RC S and V slabs. Considering that ultimate deflection cannot be calculated through the *I_e_* based deflection prediction equations provided in the current codes or standards, the approach proposed in this study will be very useful in predicting deflection in the ultimate state of flexural members.

### 4.3. Prediction of Immediate Deflections

**Figure 11:**
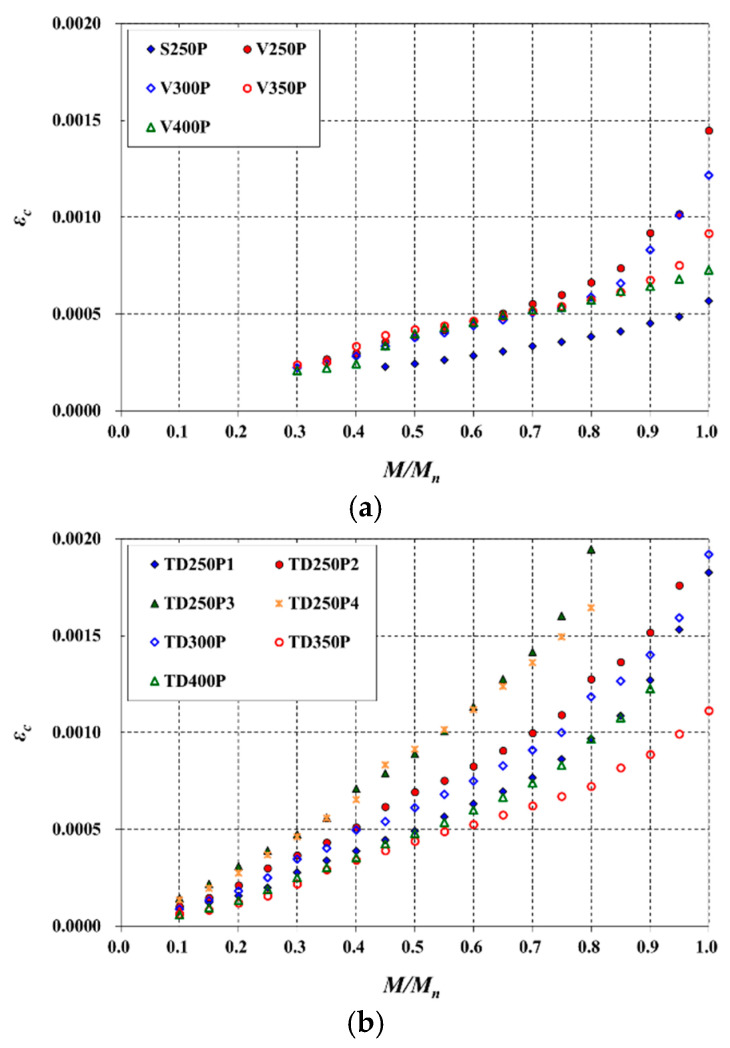
Variations of *ε_c_* with *M*/*M_n_*: (**a**) S and V slabs; (**b**) TD slabs.

Equation (12), derived in this study, has an advantage in that it can predict deflections without directly calculating *EI*. However, to calculate immediate deflections for service loads, variations of *ε_c_* and *c* must be defined according to the load level (i.e., *M*/*M_n_*). The *ε_c_* on the concrete top surface obtained in the test is plotted in Figure 11. The relationship between *ε_c_* and the *M*/*M_n_* was almost linear up to approximately 0.8 *M*/*M_n_*, and thereafter, *ε_c_* increased more rapidly. The boundary of the linear relationship exceeded the upper limit of the effective prediction range, 0.65 *M*/*M_n_*. Therefore, the *ε_c_* of the slab corresponding to the *M*/*M_n_* under service loads could be obtained from the initial slope, although the slope varies with *ρ* as clearly seen in Figure 11 [27].

Meanwhile, for the S slab and V slab under the same conditions, whether voids exist or not, a problem arises in that the predicted deflection values through Equation (12) are calculated the same as long as *a* is smaller than *t_ft_*. Therefore, the effect of stiffness reduction owing to voids must be additionally considered so that the deflection of the V slab can be evaluated as greater. At the same *M*/*M_n_*, *ε_c_* becomes larger as *ρ* increases (Figure 11), which results in a larger *δ* according to Equation (12). Focusing on this, the area of voids was subtracted from the reference area for computing the *ρ* of the V slabs. As the TD slabs also included voids, the *ρ* was calculated in the same manner. However, as in the deflection prediction based on the code modification (Section 3.4), only 50% of the steel deck area was considered to be an effective contributing area.

**Figure 12:**
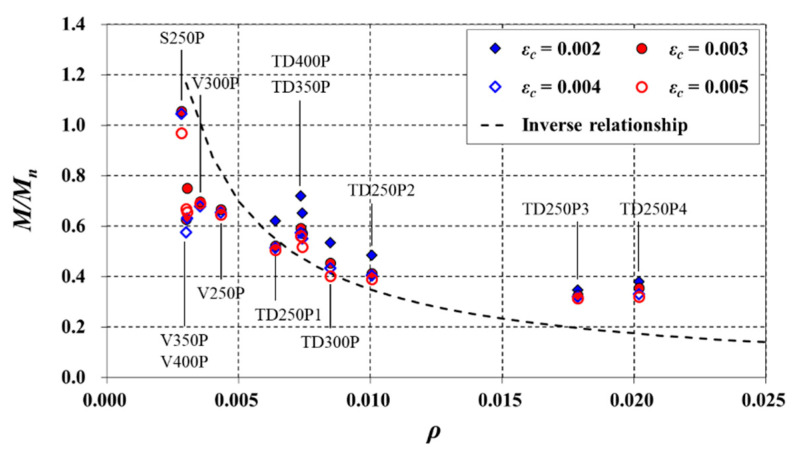
Variations of *M*/*M_n_* with *ρ*.

Under the above conditions, the relationship between the *ρ* of the slabs and *M*/*M_n_* is shown in Figure 12. In the figure, the *M*/*M_n_* values were extracted when *ε_c_* was 0.0002, 0.0003, 0.0004, and 0.0005. The TD slabs had a linear relationship between *ε_c_* and the *M*/*M_n_* at those points (see Figure 11). Overall, *M*/*M_n_* tended to decrease as *ρ* increased. Based on the aforementioned considerations, the ratio of *M*/*M_n_* to *ε_c_* (i.e., the initial slope) under the service load was fixed at 1400 when *ρ* was 0.005 [27], and it was defined to be inversely proportional to *ρ*, as in Equation (13):

(13) $$\frac{M}{M_{n}}=1400\left(\frac{0.005}{\rho }\right)\epsilon_{c}$$

**Figure 13:**
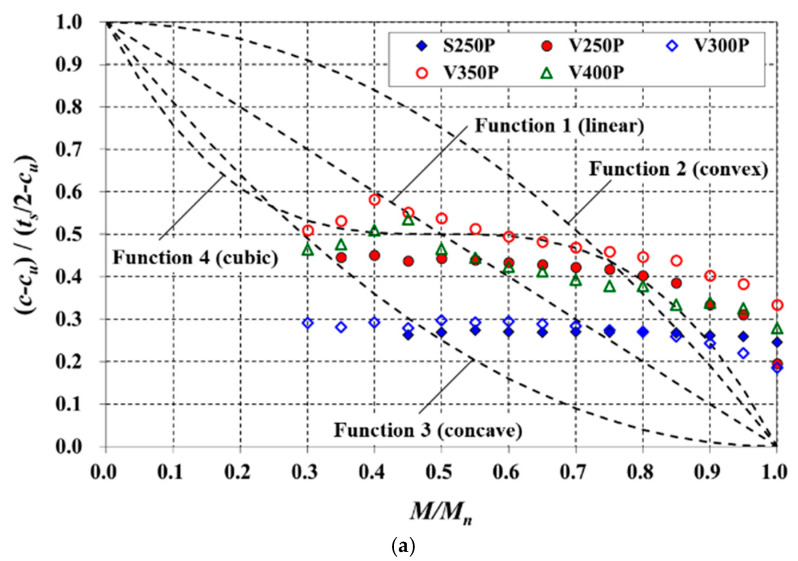

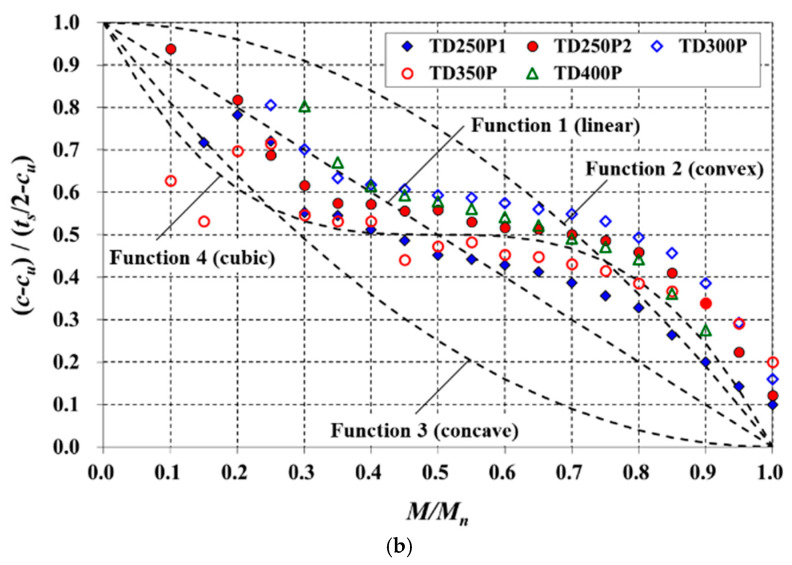
Variations of the neutral axis depth with *M*/*M_n_*: (**a**) S and V slabs; (**b**) TD slabs.

Figure 13 presents the normalized variations of the neutral axis depth with a load level. The *c* values were calculated based on a linear strain distribution using strains measured in the top and bottom reinforcing bars. The dotted lines in the figure are variation functions, which have been assumed in four forms for simplicity. As the vertical axis is (*c* −*c_u_*)/(*t_s_*/2−*c_u_*), the value converges to zero in the ultimate state (i.e., *M*/*M_n_* = 1). The value of the vertical axis for the uncracked section is not 0, but in this study, initial cracks occurred in many specimens owing to preloading (see Section 2.1). Therefore, assuming that some cracks existed at the beginning, the four functions were set to pass through the coordinate values (0, 1) and (1, 0). Function 1 was defined as a linear function, and Functions 2 and 3 were defined as concave downward and concave upward quadratic functions with vertices at (0, 1) and (1, 0), respectively. Function 4 is a cubic function that additionally passes through the coordinates (0.5, 0.5), which was set so that the derivative values had the same sign.

For the TD specimens, where the influence of preloading was insignificant, the variation of the neutral axis depth tended to be similar to Function 1 or Function 4. Conversely, for the RC S and V specimens, the *M*/*M_n_* values owing to preloading were quite large, with values between 0.252 and 0.450 (Table 1). Their (*c*−*c_u_*)/(*t_s_*/2−*c_u_*) values in the effective prediction range exhibited only a small change, whereas the *M*/*M_n_* values exhibited a large difference among specimens. Moreover, the value of *c* was less than *c_u_* when the *M_n_* was reached; hence, the values of (*c*−*c_u_*)/(*t_s_*/2−*c_u_*) did not approach 0 but were in the range of 0.186–0.334.

The comparison results for the predicted immediate deflections are shown in Table 9. In the table, values with an error between *δ_exp_* and *δ_cal_* of less than 20% (0.8 ≤ *δ_exp_*/*δ_cal_* ≤ 1.2) are underlined. Although there were differences depending on the assumed variation functions, the proposed procedure exhibited a large error and unstable prediction over the entire load level for the S and V slabs. They were very lightly reinforced slabs, and the values of *c* upon reaching *M_n_* were significantly different from those expected [9]. As the S and V slabs had a fairly low *ρ*, they were out of the main area of interest during the derivation of Equation (13), which would also increase the errors. Moreover, owing to causes such as initial cracks, the neutral axis depth demonstrated only a small difference compared with *M*/*M_n_*, as shown in Figure 13a. These factors would have resulted in less effective predictions for the S and V slabs.

This procedure was mainly derived for the TD slabs with a general range of *ρ*, and thus, much better predictions were achieved for TD slabs compared with S and V slabs. TD250P3 and TD250P4, which were designed with a very high *ρ*, were not of primary concern, and as can be expected, their predictions were quite conservative.

**Figure 14:**
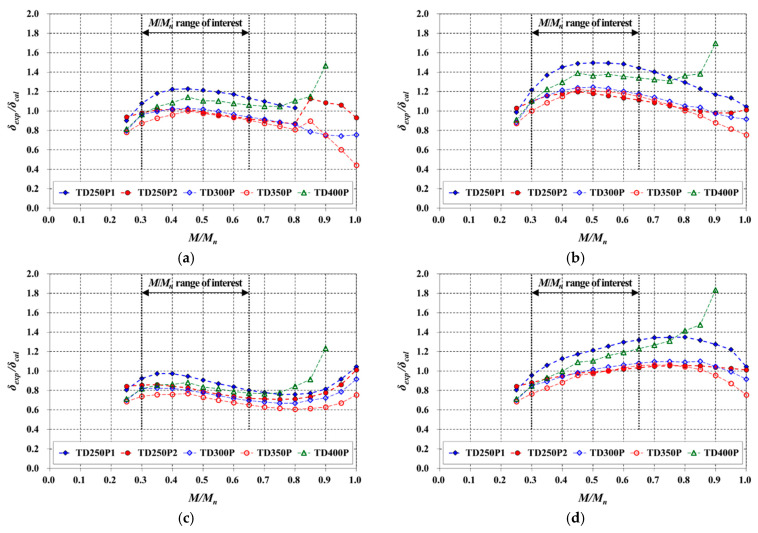
Comparison of immediate deflection predicted for the TD slabs using (**a**) Function 1 (linear); (**b**) Function 2 (convex); (**c**) Function 3 (concave); (**d**) Function 4 (cubic).

Figure 14 plots *δ_exp_*/*δ_cal_* with a load level for typical TD slabs. Within the effective prediction range (0.3 ≤ *M*/*M_n_* ≤ 0.65), Function 2 yielded nonconservative results, whereas Function 3 yielded conservative results. Functions 1 and 4, in which the variation of the neutral axis depth was similar to that of the experiment, yielded more reasonable results on average. The prediction for the TD250P1, which had the lowest *ρ* among the TD slabs, underestimated the deflection the most for all cases.

The predictions based on Function 1 exhibited a gently convex distribution of *δ_exp_*/*δ_cal_* within the effective prediction range. Conversely, when based on Function 4, *δ_exp_*/*δ_cal_* tended to increase as the *M*/*M_n_* increased. The *δ_exp_* increased as the *M*/*M_n_* increased, and accordingly, when the *δ_exp_*/*δ_cal_* values were the same, the error between the *δ_exp_* and *δ_cal_* increased at high *M*/*M_n_* values. Given these facts, it can be concluded that the results based on Function 1 gave the best predictions among the assumed variation functions.

### 4.4. Significance and Limitations

In this study, a new prediction method has been proposed to calculate immediate deflections by combining structural mechanics and flexure theory for RC members. According to the current codes or standards [11,13,24,25], predictions can be used only under the service load, but the proposed method was evaluated to be able to predict ultimate deflections as well (Section 4.2).

Instead of being able to predict the deflection without computing the empirically derived *I_e_*, the challenge still remains for the proposed method to define the variation functions for *ε_c_* and *c*. In this study, by focusing on the results of a limited number of experiments performed on composite steel deck slabs voided by circular tubes (i.e., the TD slabs), the influence of *ε_c_* was reflected indirectly and variation functions for *c* were assumed. The prediction results based on the proposed method were demonstrated to be effective (Section 4.3), but the procedure is incomplete and needs to be further improved.

If subsequent studies are carried out, it is expected that variation functions derived theoretically based on cross-sectional details can be used. The proposed procedure has important significance in presenting a new perspective in predicting the deflection of RC and composite flexural members, which is a more rational method and is a process that leads to advanced engineering design technology.

## 5. Conclusions

Focusing on TD slabs, which are composite voided slabs, research has been conducted to derive deflection prediction approaches, and for this purpose, the results of previously performed experimental tests were used. Prediction methods based on the current code modification and a new approach, which predicts deflection in a more rational way—different from existing approaches—were proposed, and the effectiveness of both methods was evaluated. The conclusions drawn from this study can be summarized as follows:

(1) For the solid slab with a low *ρ* of 0.283% (S250P), the ACI 318-14 code significantly underestimated deflection, but the deflection calculated by the ACI 318-19 code yielded improved results. The A23.3-19 standard, which uses 0.5*f_r_* to compute *M_cr_*, also predicted the deflection of S250P relatively well within the range of interest. By contrast, both concrete standards (i.e., ACI 318-19 and A23.3-19) produced nonconservative predictions for V slabs. To account for more severe cracking owing to shrinkage and temperature effects on the V slabs, a modification was made to compute the *M_cr_* using 0.35*f_r_* in the CSA A23.3-19 standard (or ACI 318-14 code). The modified equation could effectively reflect the stiffness reduction owing to voids, and accordingly, the predicted *δ_cal_* became more acceptable within the range of interest.
(2) The *I_e_* equation given in the CSA A23.3-19 standard and ACI 318-14 code was also valid in calculating the immediate deflection of the TD slabs. However, in order not to overestimate the flexural stiffness of the member, it was recommended to compute *I_cr_* by considering only half the area of the steel deck as the effective contributing area. The suggested prediction method based on code modification could be practically used to calculate the deflection of the TD slabs.
(3) Using the suggested method based on the code modification, the comparative performance of the S slabs and TD slabs for immediate and long-term deflections was evaluated. Calculations were made for cases where both types of slabs had the same level of flexural strength and the same tension reinforcing bars. Deflection *δ_L_* tended to occur largely in TD slabs, whereas deflection *δ_cp_*_+*sh*_ + *δ_L_* was predicted to be smaller under similar slabs. Moreover, the maximum *l* of the TD slabs was calculated to be at least 10% larger than that of the S slabs. The results demonstrated that the TD slab system is more efficient in deflection control compared with the S slab.
(4) Combining structural mechanics and flexure theory for RC members, a new prediction method has been proposed to calculate deflections without computing the empirically derived *I_e_*. The predicted *δ_cal_* using the proposed method had a considerably large error with the *δ_exp_* at *M_n_* and was more closely correlated with the deflection at the *M_max_*. Considering that the ultimate deflection cannot be calculated using the prediction equations according to the current standards, the proposed approach will be especially useful in predicting deflection in the ultimate state of flexural members.
(5) A computation of *EI* was not required to calculate immediate deflections using the new prediction method, but the variations of *ε_c_* and *c* with the load level needed to be defined. Based on the experimental results performed, the ratio of *M*/*M_n_* to *ε_c_* was defined to be inversely proportional to *ρ*, and four variation functions were assumed for the neutral axis depth. For typical TD slabs, Function 1 (linear) and Function 4 (cubic) yielded reasonable results, and Function 1 gave the best predictions.

## Figures and Tables

**Table 1:** List of tested slab specimens under consideration.

Specimen	*t_s_* (mm)	*f′_c_* (MPa)	Void Arrangement				Bottom Reinforcement		*M_pre_*/*M_n_*
			*D_e_* (mm)	*n_v_*	*A_v_* (mm^2^)	*R_v_* (%)	Long.	Transv.	
S250P	250	25.94	-	-	-	-	6-D13	D10@250	0.450
V250P	250	22.03	140	6	92,363	30.79	6-D13	D10@250	0.318
TD250P1	250	25.94	140	6	92,363	30.79	-	-	0.127
TD250P2	250	25.94	140	6	92,363	30.79	6-D13	-	0.094
TD250P3	250	25.94	140	6	92,363	30.79	12-D16	-	0.063
TD250P4	250	22.03	140	6	92,363	30.79	10-D19	-	0.065
V300P	300	25.94	190	4	113,411	31.50	6-D13	D10@250	0.303
TD300P	300	25.94	190	4	113,411	31.50	6-D13	-	0.090
V350P	350	24.55	240	3	135,717	32.31	6-D13	D10@250	0.293
TD350P	350	24.55	240	3	135,717	32.31	6-D13	-	0.088
V400P	400	20.47	290	3	198,156	41.28	6-D13	D10@250	0.252
TD400P	400	20.47	290	3	198,156	41.28	6-D13	-	0.076

Note: For steel reinforcement, “D” means deformed bar as specified in KS D 3504 [22]. *A_v_* is the area of voids. *M_pre_* and *M_n_* denote the maximum bending moment due to preloading and the nominal flexural strength, respectively.

**Table 2:** Serviceability limit states (SLS) for deflection checks.

*t_s_* (mm)	*R_v_* (%)	*D* (kPa)			*L* (kPa)	Loads for Limit States (kPa)				SLS/(ULS/*φ*)		
		SW	SDL	Sum		ULS	SLS 1	SLS 2	SLS 3	SLS 1	SLS 2	SLS 3
250	-	6.00	2.50	8.50	6.0	19.80	8.50	11.50	14.50	0.386	0.523	0.659
250	30.79	4.15	2.50	6.65	6.0	17.58	6.65	9.65	12.65	0.341	0.494	0.648
300	31.50	4.93	2.50	7.43	6.0	18.52	7.43	10.43	13.43	0.361	0.507	0.653
350	32.31	5.69	2.50	8.19	6.0	19.42	8.19	11.19	14.19	0.379	0.518	0.657
400	41.28	5.64	2.50	8.14	6.0	19.36	8.14	11.14	14.14	0.378	0.518	0.657

**Table 3:** Values of parameters used to calculate immediate deflection (V slabs).

Specimen	*f_r_* (MPa)	*E_c_* (MPa)	*y_t_* (mm)	*I_g_* (mm^4^)	*I_cr_* (mm^4^)	*I_g_*/*I_cr_*	*M_cr_* (kN∙m)	
							0.5*f_r_*	0.35*f_r_*
V250P	2.910	22,059	127.2	1.446 × 10^9^	2.561 × 10^8^	5.646	16.54	11.58
V300P	3.157	23,936	152.3	2.440 × 10^9^	3.672 × 10^8^	6.645	25.29	17.70
V350P	3.072	23,288	177.4	3.794 × 10^9^	5.372 × 10^8^	7.063	32.85	23.00
V400P	2.805	21,266	203.5	5.350 × 10^9^	7.890 × 10^8^	6.781	36.87	25.81

**Table 4:** Values of parameters used to calculate immediate deflection (TD slabs).

Specimen	*f_r_* (MPa)	*E_c_* (MPa)	*y_t_* (mm)	*I_g_* (mm^4^)	Full Contribution of *A_sd_*		Half Contribution of *A_sd_*	
					*I_cr_* (mm^4^)	*I_g_*/*I_cr_*	*I_cr_* (mm^4^)	*I_g_*/*I_cr_*
TD250P2	3.157	23,936	127.2	1.446 × 10^9^	8.456 × 10^8^	1.710	5.779 × 10^8^	2.502
TD300P	3.157	23,936	152.3	2.440 × 10^9^	1.318 × 10^9^	1.851	8.940 × 10^8^	2.729
TD350P	3.072	23,288	177.4	3.794 × 10^9^	1.941 × 10^9^	1.955	1.312 × 10^9^	2.892
TD400P	2.805	21,266	203.5	5.350 × 10^9^	2.839 × 10^9^	1.884	1.921 × 10^9^	2.785

**Table 5:** Serviceability limit states (SLS) for deflection checks.

Specimen	SLS 1		SLS 2		SLS 3		SLS 3 SLS 1	
	*δ_exp_* (mm)	*l*/*δ_exp_*	*δ_exp_* (mm)	*l*/*δ_exp_*	*δ_exp_* (mm)	*l*/*δ_exp_*	*δ_exp_* (mm)	*l*/*δ_exp_*
TD250P1	11.41	526	20.90	287	31.71	189	20.30	296
TD250P2	15.06	399	25.06	239	36.01	167	20.99	286
TD250P3	17.79	337	29.81	201	43.52	138	25.73	233
TD250P4	18.39	326	29.37	204	41.22	146	22.83	263
TD300P	11.83	507	19.77	304	28.25	212	16.42	365
TD350P	9.40	638	15.13	397	21.36	281	11.96	502
TD400P	9.28	647	15.20	395	22.04	272	12.76	470

**Table 6:** Deflection comparison of TD slabs and S slabs with the same strength level.

*t_s_* (mm)	*l* (m)	*l/t_s_*	Slab Type	Longitudinal Bar		Parameter		Deflection (mm)	
				Top	Bottom	*I_cr_* (mm^4^)	*λ_δ_*	*δ_L_*	*δ_cp+sh_* + *δ_L_*
250	5.0	20.0	S	5-D10	10-D16	5.347 × 10^8^	1.875	4.64 (*l*/1076)	12.04 (*l*/415)
			TD	5-D10	-	4.274 × 10^8^	1.842	4.59 (*l*/1089)	10.34 (*l*/484)
	5.25	21.0	S	5-D10	10-D16	5.347 × 10^8^	1.875	6.59 (*l*/797)	17.09 (*l*/307)
			TD	5-D10	-	4.274 × 10^8^	1.842	6.68 (*l*/786)	15.11 (*l*/347)
	5.5	22.0	S	5-D10	10-D16	5.347 × 10^8^	1.875	8.81 (*l*/624)	23.20 (*l*/237)
			TD	5-D10	-	4.274 × 10^8^	1.842	9.25 (*l*/595)	21.16 (*l*/260)
	5.75	23.0	S	5-D10	10-D16	5.347 × 10^8^	1.875	10.77 (*l*/534)	29.86 (*l*/193)
			TD	5-D10	-	4.274 × 10^8^	1.842	12.30 (*l*/467)	28.59 (*l*/201)
300	6.0	20.0	S	5-D10	10-D16	8.382 × 10^8^	1.896	7.16 (*l*/838)	19.05 (*l*/315)
			TD	5-D10	-	6.532 × 10^8^	1.865	7.23 (*l*/830)	16.71 (*l*/359)
	6.3	21.0	S	5-D10	10-D16	8.382 × 10^8^	1.896	9.57 (*l*/658)	26.27 (*l*/240)
			TD	5-D10	-	6.532 × 10^8^	1.865	10.34 (*l*/609)	24.09 (*l*/261)
	6.6	22.0	S	5-D10	10-D16	8.382 × 10^8^	1.896	11.91 (*l*/554)	34.53 (*l*/191)
			TD	5-D10	-	6.532 × 10^8^	1.865	14.14 (*l*/467)	33.36 (*l*/198)
350	7.0	20.0	S	6-D10	10-D16	1.214 × 10^9^	1.895	9.86 (*l*/710)	27.73 (*l*/252)
			TD	6-D10	-	9.290 × 10^8^	1.860	10.73 (*l*/652)	25.21 (*l*/278)
	7.35	21.0	S	6-D10	10-D16	1.214 × 10^9^	1.895	12.53 (*l*/586)	37.37 (*l*/197)
			TD	6-D10	-	9.290 × 10^8^	1.860	14.95 (*l*/492)	35.72 (*l*/206)
400	8.0	20.0	S	8-D10	12-D16	1.931 × 10^9^	1.880	11.15 (*l*/717)	35.22 (*l*/227)
			TD	8-D10	4-D10	1.470 × 10^9^	1.816	13.16 (*l*/608)	31.37 (*l*/255)
	8.4	21.0	S	8-D10	12-D16	1.931 × 10^9^	1.880	13.80 (*l*/609)	46.46 (*l*/181)
			TD	8-D10	4-D10	1.470 × 10^9^	1.816	17.16 (*l*/489)	42.70 (*l*/197)

Note: The values were calculated for a width of 1.2 m.

**Table 7:** Deflection comparison of TD slabs and S slabs with the same tension reinforcing bars.

*t_s_* (mm)	Bottom Bar	Slab Type	Strength			*I_cr_* (mm^4^)	*l* (m)	Deflection (mm)	
			*M_u_* (kN∙m)	*φM_n_* (kN∙m)	*φM_n_*/*M_u_*			*δ_L_*	*δ_cp+sh_* + *δ_L_*
250	8-D13	S	77.25	78.53	1.02	3.150 × 10^8^	5.10	7.12 (*l*/716)	16.37 (*l*/312)
		TD	88.73	206.89	2.33	6.466 × 10^8^	5.80	9.03 (*l*/642)	23.69 (*l*/245)
	10-D13	S	83.43	97.22	1.17	3.790 × 10^8^	5.30	8.80 (*l*/602)	20.97 (*l*/253)
		TD	90.26	223.16	2.47	6.955 × 10^8^	5.85	8.78 (*l*/666)	23.66 (*l*/247)
	12-D13	S	85.01	115.53	1.36	4.394 × 10^8^	5.35	8.59 (*l*/623)	21.21 (*l*/252)
		TD	93.37	239.04	2.56	7.425 × 10^8^	5.95	8.80 (*l*/676)	24.59 (*l*/242)
	10-D16	S	88.22	147.12	1.67	5.347 × 10^8^	5.45	8.42 (*l*/647)	21.96 (*l*/248)
		TD	96.54	266.18	2.76	8.170 × 10^8^	6.05	8.38 (*l*/722)	24.69 (*l*/245)
	12-D16	S	91.48	173.76	1.90	6.151 × 10^8^	5.55	8.13 (*l*/682)	22.58 (*l*/246)
		TD	101.39	289.02	2.85	8.812 × 10^8^	6.20	8.32 (*l*/745)	25.86 (*l*/240)
300	10-D13	S	118.55	120.03	1.01	5.876 × 10^8^	6.10	10.03 (*l*/608)	24.57 (*l*/248)
		TD	124.69	275.24	2.21	1.083 × 10^9^	6.70	9.83 (*l*/682)	27.17 (*l*/247)
	12-D13	S	120.50	142.90	1.19	6.834 × 10^8^	6.15	9.72 (*l*/633)	24.67 (*l*/249)
		TD	128.44	295.69	2.30	1.159 × 10^9^	6.80	9.70 (*l*/701)	27.93 (*l*/243)
	10-D16	S	126.45	182.87	1.45	8.382 × 10^8^	6.30	9.57 (*l*/658)	26.27 (*l*/240)
		TD	134.17	331.20	2.47	1.283 × 10^9^	6.95	9.39 (*l*/740)	28.75 (*l*/242)
	12-D16	S	130.50	216.66	1.66	9.678 × 10^8^	6.40	9.14 (*l*/700)	26.68 (*l*/240)
		TD	138.06	361.19	2.62	1.388 × 10^9^	7.05	8.99 (*l*/784)	28.81 (*l*/245)
	10-D19	S	134.61	254.85	1.89	1.101 × 10^9^	6.50	8.64 (*l*/752)	26.84 (*l*/242)
		TD	144.00	394.77	2.74	1.496 × 10^9^	7.20	8.81 (*l*/817)	29.68 (*l*/243)
350	10-D16	S	169.09	216.96	1.28	1.214 × 10^9^	7.05	10.22 (*l*/690)	29.00 (*l*/243)
		TD	177.25	390.74	2.20	1.861 × 10^9^	7.80	10.36 (*l*/753)	32.51 (*l*/240)
	12-D16	S	173.92	257.17	1.48	1.406 × 10^9^	7.15	9.74 (*l*/734)	29.32 (*l*/244)
		TD	181.83	426.61	2.35	2.019 × 10^9^	7.90	9.86 (*l*/801)	32.37 (*l*/244)
	10-D19	S	181.29	302.97	1.67	1.608 × 10^9^	7.30	9.43 (*l*/774)	30.46 (*l*/240)
		TD	188.80	467.14	2.47	2.186 × 10^9^	8.05	9.55 (*l*/843)	33.00 (*l*/244)
	12-D19	S	188.82	356.95	1.89	1.850 × 10^9^	7.45	8.85 (*l*/842)	30.89 (*l*/241)
		TD	198.29	514.85	2.60	2.386 × 10^9^	8.25	9.30 (*l*/887)	34.02 (*l*/242)
400	10-D19	S	231.55	357.99	1.55	2.217 × 10^9^	8.00	9.85 (*l*/812)	32.53 (*l*/246)
		TD	237.90	556.47	2.34	3.008 × 10^9^	9.05	11.07 (*l*/817)	37.58 (*l*/241)
	12-D19	S	243.27	423.80	1.74	2.557 × 10^9^	8.20	9.45 (*l*/868)	33.93 (*l*/242)
		TD	248.53	616.79	2.48	3.290 × 10^9^	9.25	10.67 (*l*/867)	38.21 (*l*/242)
	10-D22	S	249.24	470.15	1.89	2.776 × 10^9^	8.30	9.05 (*l*/917)	34.07 (*l*/244)
		TD	256.65	659.00	2.57	3.468 × 10^9^	9.40	10.54 (*l*/892)	38.99 (*l*/241)

Note: The values were calculated for a width of 1.2 m, and the compression reinforcements at the top were 5-D10 for all cases.

**Table 8:** Calculated ultimate deflections.

Specimen	*M_n_* (kN∙m)	*M_max_* (kN∙m)	*c_u_* (mm)	*κ* (mm^−1^)	*I_e_* (mm^4^)	*δ_cal_* (mm)	*M_n_*		*M_max_*		Remarks
							*δ_exp_* (mm)	*δ_exp_*/*δ_cal_*	*δ_exp_* (mm)	*δ_exp_*/*δ_cal_*	
S250P	72.71	97.06	14.89	2.015 × 10^−4^	1.507 × 10^7^	400.21	47.15	0.118	402.71	1.006	No peak
V250P	72.33	90.32	17.53	1.711 × 10^−4^	1.916 × 10^7^	417.93	89.23	0.214	520.16	1.245	No peak
V300P	89.44	114.38	14.89	2.015 × 10^−4^	1.855 × 10^7^	499.96	80.50	0.161	482.63	0.965	-
V350P	106.06	138.32	15.73	1.907 × 10^−4^	2.388 × 10^7^	479.59	50.39	0.105	328.83	0.686	Bar fracture
V400P	122.36	173.57	18.86	1.591 × 10^−4^	3.617 × 10^7^	422.45	27.53	0.065	279.02	0.660	Bar fracture
TD250P1	181.79	216.07	34.49	8.699 × 10^−5^	8.731 × 10^7^	271.53	88.09	0.324	307.48	1.132	-
TD250P2	244.68	273.03	49.38	6.076 × 10^−5^	1.682 × 10^8^	197.42	97.07	0.492	216.82	1.098	-
TD250P3	363.61	312.24	85.26	3.519 × 10^−5^	4.317 × 10^8^	119.73	-	-	75.34	0.629	Early failure
TD250P4	351.77	284.88	131.64	2.279 × 10^−5^	6.997 × 10^8^	77.83	-	-	58.03	0.746	Early failure
TD300P	300.19	343.70	49.38	6.076 × 10^−5^	2.064 × 10^8^	197.40	73.84	0.374	225.34	1.142	-
TD350P	354.39	430.52	52.16	5.752 × 10^−5^	2.646 × 10^8^	187.19	50.10	0.268	211.38	1.129	-
TD400P	405.00	364.42	62.55	4.796 × 10^−5^	3.971 × 10^8^	158.01	-	-	61.29	0.388	Early failure

Note: In the table, *I_e_* represents *I* equivalent calculated as *Mc*/*E_c_ε_c_* using Equation (10).

**Table 9:** Comparison of immediate deflection (*δ_exp_*/*δ_cal_*).

Function	Specimen	*M*/*M_n_*											
		0.25	0.30	0.35	0.40	0.45	0.50	0.55	0.60	0.65	0.70	0.75	0.80
1	S250P	-	-	-	-	0.999	0.998	1.114	1.175	1.282	1.292	1.231	1.203
	V250P	-	-	0.971	0.975	1.239	1.445	1.426	1.421	1.393	1.298	1.204	1.132
	V300P	-	1.157	1.450	1.665	1.933	1.858	1.793	1.712	1.580	1.448	1.309	1.216
	V350P	-	1.044	1.112	1.620	1.903	1.839	1.832	1.803	1.658	1.531	1.378	1.229
	V400P	1.043	0.795	0.734	0.921	1.279	1.364	1.340	1.384	1.290	1.216	1.179	1.029
	TD250P1	0.902	1.078	1.181	1.224	1.229	1.213	1.193	1.171	1.131	1.098	1.058	1.032
	TD250P2	0.940	0.980	1.012	1.016	1.017	0.989	0.963	0.941	0.919	0.902	0.883	0.870
	TD250P3	0.706	0.711	0.722	0.744	0.761	0.761	0.766	0.779	0.780	0.796	0.822	0.851
	TD250P4	0.715	0.749	0.775	0.788	0.823	0.824	0.833	0.842	0.860	0.885	0.910	0.954
	TD300P	0.795	0.965	0.996	1.022	1.026	1.016	0.994	0.964	0.939	0.913	0.885	0.862
	TD350P	0.782	0.875	0.927	0.960	1.001	0.975	0.953	0.933	0.907	0.872	0.843	0.808
	TD400P	0.814	0.967	1.045	1.086	1.143	1.105	1.104	1.078	1.061	1.049	1.046	1.106
2	S250P	-	-	-	-	1.000	1.032	1.193	1.305	1.475	1.539	1.513	1.516
	V250P	-	-	1.003	1.054	1.398	1.696	1.733	1.784	1.798	1.714	1.616	1.529
	V300P	-	1.152	1.532	1.852	2.254	2.260	2.268	2.243	2.136	2.009	1.854	1.741
	V350P	-	1.053	1.186	1.816	2.232	2.251	2.332	2.379	2.259	2.144	1.972	1.782
	V400P	1.040	0.851	0.831	1.093	1.584	1.755	1.784	1.900	1.818	1.753	1.726	1.517
	TD250P1	0.987	1.215	1.367	1.451	1.488	1.496	1.494	1.482	1.442	1.403	1.345	1.296
	TD250P2	1.030	1.098	1.156	1.180	1.199	1.178	1.157	1.136	1.112	1.087	1.055	1.025
	TD250P3	0.743	0.756	0.773	0.802	0.825	0.827	0.834	0.848	0.847	0.861	0.883	0.907
	TD250P4	0.715	0.749	0.775	0.788	0.823	0.824	0.833	0.842	0.860	0.885	0.910	0.954
	TD300P	0.883	1.098	1.159	1.214	1.239	1.244	1.231	1.202	1.174	1.141	1.097	1.052
	TD350P	0.872	1.001	1.086	1.149	1.221	1.208	1.195	1.180	1.153	1.107	1.064	1.005
	TD400P	0.909	1.107	1.223	1.298	1.389	1.364	1.378	1.357	1.340	1.323	1.310	1.363
3	S250P	-	-	-	-	0.999	0.927	0.957	0.932	0.941	0.886	0.800	0.760
	V250P	-	-	0.920	0.852	1.003	1.089	1.005	0.943	0.879	0.788	0.717	0.678
	V300P	-	1.164	1.319	1.382	1.473	1.304	1.164	1.033	0.893	0.776	0.678	0.625
	V350P	-	1.029	0.997	1.328	1.431	1.273	1.170	1.066	0.915	0.797	0.689	0.607
	V400P	1.048	0.715	0.603	0.698	0.897	0.888	0.812	0.784	0.688	0.618	0.581	0.506
	TD250P1	0.806	0.924	0.974	0.973	0.945	0.906	0.870	0.838	0.802	0.778	0.759	0.760
	TD250P2	0.843	0.854	0.860	0.843	0.827	0.791	0.761	0.738	0.721	0.712	0.707	0.713
	TD250P3	0.668	0.665	0.669	0.684	0.697	0.694	0.698	0.709	0.713	0.731	0.760	0.795
	TD250P4	0.715	0.749	0.775	0.788	0.823	0.824	0.833	0.842	0.860	0.885	0.910	0.954
	TD300P	0.700	0.822	0.822	0.820	0.802	0.777	0.748	0.717	0.696	0.680	0.669	0.668
	TD350P	0.685	0.739	0.757	0.759	0.769	0.731	0.700	0.675	0.652	0.629	0.617	0.607
	TD400P	0.712	0.817	0.855	0.863	0.883	0.835	0.818	0.790	0.773	0.767	0.777	0.843
4	S250P	-	-	-	-	0.999	0.998	1.133	1.236	1.408	1.498	1.513	1.568
	V250P	-	-	0.944	0.933	1.199	1.445	1.494	1.577	1.647	1.637	1.616	1.603
	V300P	-	1.162	1.380	1.567	1.855	1.858	1.898	1.939	1.927	1.904	1.854	1.840
	V350P	-	1.033	1.050	1.519	1.822	1.839	1.943	2.050	2.033	2.030	1.972	1.886
	V400P	1.048	0.734	0.661	0.839	1.210	1.364	1.435	1.600	1.615	1.650	1.726	1.611
	TD250P1	0.806	0.956	1.060	1.127	1.174	1.213	1.255	1.298	1.320	1.343	1.345	1.347
	TD250P2	0.843	0.880	0.922	0.948	0.980	0.989	1.002	1.019	1.035	1.050	1.055	1.056
	TD250P3	0.668	0.674	0.690	0.720	0.748	0.761	0.780	0.806	0.820	0.848	0.883	0.918
	TD250P4	0.715	0.749	0.775	0.788	0.823	0.824	0.833	0.842	0.860	0.885	0.910	0.954
	TD300P	0.700	0.851	0.893	0.943	0.982	1.016	1.042	1.060	1.081	1.096	1.097	1.090
	TD350P	0.685	0.767	0.826	0.881	0.956	0.975	1.002	1.033	1.056	1.061	1.064	1.044
	TD400P	0.712	0.848	0.932	0.998	1.092	1.105	1.159	1.191	1.230	1.269	1.310	1.414

## Data Availability

The data presented in this study are available on request from the corresponding author. The data are not publicly available due to privacy.
